# Bidirectional Scattering Distribution Function (BSDF): A Systematized Bibliography

**DOI:** 10.6028/jres.096.010

**Published:** 1991

**Authors:** Clara Asmail

**Affiliations:** National Institute of Standards and Technology, Gaithersburg, MD 20899

**Keywords:** bidirectional reflectance distribution function (BRDF), bidirectional scattering distribution function (BSDF), bidirectional transmittance distribution function (BTDF), coherence, diffuse reflectance, instrument signature, inverse scattering problem, polarization, scattering theory, specular reflectance, stray light, surface finish

## Abstract

In conjunction with the development of a bidirectional scattering metrology project, a large number of papers pertaining to the theory and measurement of bidirectional scattering from optical surfaces were collected and categorized. This collection includes papers that deal with various aspects of the bidirectional scattering distribution function (BSDF), its measurement, interpretation, use, and implications. Each paper is classified in one or more subject categories on the basis of its technical content. The subject categories are included just to serve as a key to the most salient characteristics of each paper cited. Because of the interest in this field, this bibliography is being published as a service to the public.

## 1. Introduction

The bidirectional scattering distribution function (BSDF) radiometrically characterizes the scatter of optical radiation from a surface as a function of the angular positions of the incident and scattered beams. By definition, it is the ratio of the scattered radiance to the incident irradiance: the unit is inverse steradian. The term bidirectional reflectance distribution function (BRDF) is used when specifically referring to reflected scatter. Likewise, bidirectional transmittance distribution function (BTDF) refers to scatter transmitted through a material.

The bidirectional characterization of elastic scatter from surfaces is a property that is required for the evaluation of elements contained within larger systems that need minimal or controlled scattered light. The need for this information is readily seen in applications such as ring laser gyroscopes and telescopes. This type of information is also requisite for characterization of materials intended for use in temperature control where thermal radiation must be modelled or in imaging applications where stray light must be suppressed. It may also be used to assist the acceptance/rejection process in optical manufacturing settings.

The present state of most of the facilities measuring this quantity needs to be upgraded to support new and more stringent requirements as well as recent strides in the production of high-quality optics.

There is a lack of uniformity throughout the community for physical standards which can ascertain the accuracy of BSDF measurements (paper 91). The NIST Bidirectional Scattering Metrology Project is currently developing an instrument which will later serve to develop standard reference materials as well as a standard measurement technique.

In conjunction with the development of this BSDF instrument, a large number of papers pertaining to the theory and measurement of bidirectional scattering from optical surfaces were collected and categorized. This collection includes papers that deal with various aspects of the BSDF, its measurement, interpretation, use, and implications.

Each paper is classified in one or more subject categories on the basis of its technical content. The subject categories are included just to serve as a key to the most salient characteristics of each paper cited. In the literature, there is a bibliography (paper 117) of papers published prior to 1975 that relate to scattering from surfaces. Building upon that bibliography, this bibliography includes papers related to BSDF published since that time to the present. Neither the category classification list nor the list of papers is complete. There were two selection criteria used to determine whether a paper should be included in this bibliography: the paper was used during certain phases of the NIST project development and/or it was regarded as relevant and important to the field. However, inclusion or omission from the list does not necessarily imply endorsement or reproval, respectively. This systematized bibliography is sufficiently extensive to be of significant help to workers in the field, and particularly to those just beginning to work in it. For further breadth of information, the reader is suggested to review the conference proceedings from the Society of Photo-Optical Instrumentation Engineers (SPIE) that focus on scattering from surfaces; some papers from each of these are cited here.

## 2. Categories

As an aid to identifying papers related to a certain field, each of the papers is listed under each category in which it has been classified. Due to the wide interdisciplinary nature of optical scattering metrology, some topics had to be grouped together so that the list would not become unmanageable. (In many instances, the title of the articles suggest the information covered.) The subject categories are coded and used as follows:

### Abs–Aberrations

The optical design of a system determines the aberrations that will be present – assuming accurate alignment–and their magnitudes. The calculation of these aberrations and their effect on the measurement of BSDF are addressed in the following papers: 3,96,102,159,160.

### Aprt–Apparatus

Many different instruments are described in the literature. A sampling of these apparatus overviews is contained in the following papers: 4,12,31,41,51,52,63,65,66,76,77,79,83,84,87,89,90,93,94,95,103,111,112,132,113,118,120,122,128,129,136,137,138,140,145,146,151,159,160,161,166,168,169.

### Calb–Calibration

Techniques for calibration and/or error analysis include reference methods and absolute methods. Specific details concerning calibration and the philosophy of calibration are discussed in these papers: 23,33,34,83,89,108,145,158.

### Cob–Coherence

The use of laser sources in BSDF instruments is very common. Coherence becomes a critical property of these sources in diffuse BSDF metrology. The following papers address the property as it pertains to radiometry in general and to BSDF metrology in particular: 3,8,37,39,40,49,59,61,63,73,80,97,98,101,125,126.

### Desn–Design

Design criteria and plans or layouts of components or subsystems of BSDF instruments are separated out and described in detail. These subsystems include source, sample manipulation, receiver, attenuation, apertures, etc: 3,10,11,21,22,27,28,29,31,35,41,51,52,60,66,68,69,71,76,85,86,87,89,90,93,94,95,108,112,132,118,119,124,128,130,136,141,142,156,157,159,160,162,163,166,168,170.

### Diff–Diffraction

Truncation of optical beams by apertures cause diffraction and thereby affect the instrument signature of BSDF instruments. This effect is discussed in the following papers: 3,49,69,70,72,89,96,139,159,160.

### Expt–Experimental Data

These articles include experimental results in the form of tabulated data or graphs from a variety of different types of measurements. Some of these include data from actual BSDF scans on particular material samples while others give interpretive results highlighting instrument capabilities. Still other papers give profilometry data that yield topographic information. The particular type of experimental data given within each paper should be obvious from the title of the paper: 4,5,7,9,13,15,16,17,18,20,22,24,25,26,28,29,30,32,35,36,38,40,41,42,44,46,48,52,53,54,57,59,64,67,73,76,78,79,83,88,91,92,94,99,100,101,103,104,109,110,111,132,113,114,115,119,120,121,122,123,127,128,129,130,131,133,134,135,138,139,140,142,143,145,147,149,151,152,154,158,161,165,167,168,169,170.

### Inst–Instrument Signature

The background measurement of the noise equivalent BSDF, or the instrument signature, limits an instrument to the measurement of samples that have a BSDF larger than the NEBSDF. Specific instrument profiles or signatures are displayed in some of the following papers. The other papers address general concerns in obtaining and improving the signature of an instrument: 4,12,36,76,77,85,86,90,120,124,130,136,137,139,141,151,159,160,162,164,166,168,171.

### Matl–Materials/Coatings

Particular optical materials and/or coatings are identified and experimental BSDF data are given for each of these in the following papers: 2,7,9,13,16,17,22,24,25,28,29,32,35,42,48,52,53,64,67,76,78,83,92,94,99,100,104,109,110,113,114,115,121,122,123,129,130,131,133,135,138,145,149158,162,165,

### Polarization

Polarization control, theory, and associated problems are examined: 5,6,23,24,25,59,79,95,144,147,167.

### Prof–Profile Analysis Techniques

Various types of mechanical and optical surface profiling techniques are described and/or compared against optical scattering predictions of surface finish (or the inverse: predictions of optical scatter from surface finish): 1,2,17,20,30,39,43,44,46,54,56,57,59,65,88,91,100,109,111,134,137,140,150,151.

### Stnd–Standard Reference Methods

The following papers describe techniques used for, or problems in, referencing BSDF measurements, thereby assigning a level of confidence to the accuracy of the measurement: 33,53,83,91,92,108,130,145,158,164.

### Stra–Stray Light

Control, or suppression, of geometrically stray light is examined in this group of papers. Included in these are some papers which evaluate baffling materials with BRDF data: 18,22,27,28,29,42,50,58,60,69,71,78,87,90,113,123,146,149,156,157,163,170.

### Surv–Survey

Below is a list of papers that are outstanding in their fields and provide comprehensive coverage of a well-defined topic within BSDF metrology: 19,24,45,46,65,74,76,77,81,82,89,91,92,105,106,107,108,117,130,140,148,171.

### Thry–Scattering Theory

Among the theoretical questions explored throughout these papers are: basic definition of BSDF, scattering theory of surfaces, mathematical treatment of surfaces, subsurface contributions to scatter, inverse scattering problem, and scaling of BSDF with respect to angle of incidence and wave-length: 1,3,5,6,7,8,12,14,15,16,17,18,20,23,26,37,38,39,40,43,44,45,46,47,49,55,56,57,58,59,61,62,69,70,71,72,74,75,77,79,80,81,82,90,94,96,97,98,101,103,105,106,107,108,109,110,111,116,120,124,125,126,129,131,134,139,144,146,147,148,150,152,153,154,155,156,157,161,162,167,170.

### Topo–Topography/Surface Finish

Surface roughness and sample isotropy, homogeneity, and cleanliness are all topographic contributions to optical scatter. These topics as well as some subsurface contributions are treated in the following papers: 1,2,4,9,14,15,16,17,18,19,20,30,38,39,40,43,44,45,46,47,54,55,56,57,62,74,75,88,100,109,110,111,116,120,121,125,127,134,135,139,142,144,147,150,152,153,154,155,161,162,167,169.

### Tran–Transmitted Scatter

Issues related to the bidirectional transmittance distribution function, BTDF, are considered in these papers: 52,103,122,126.
